# Genome-wide analysis of antisense transcription with Affymetrix exon array

**DOI:** 10.1186/1471-2164-9-27

**Published:** 2008-01-22

**Authors:** Xijin Ge, Wendy S Rubinstein, Yong-chul Jung, Qingfa Wu

**Affiliations:** 1 Department of Mathematics and Statistics, South Dakota State University, Box 2220, Brookings, SD 57007, USA; 2 Center for Medical Genetics, Evanston Northwestern Healthcare, 1000 Central Street, Suite 620, Evanston, IL 60201, USA; 3 Center for Functional Genomics, ENH Research Institute, 1001 University Place, Evanston, IL 60201, USA; 4 Dept. of Plant Pathology & Microbiology, College of Natural and Agricultural Sciences, University of California Riverside, Riverside, CA 92521, USA; 5 Northwestern University Feinberg School of Medicine, Chicago, IL 60611, USA

## Abstract

**Background:**

A large number of natural antisense transcripts have been identified in human and mouse genomes. Study of their potential functions clearly requires cost-efficient method for expression analysis.

**Results:**

Here we show that Affymetrix Exon arrays, which were designed to detect conventional transcripts in the sense orientation, can be used to monitor antisense expression across all exonic loci in mammalian genomes. Through modification of the cDNA synthesis protocol, we labeled single-strand cDNA in the reverse orientation as in the standard protocol, thus enabling the detection of antisense transcripts using the same array. Applying this technique to human Jurkat cells, we identified antisense transcription at 2,088 exonic loci of 1,516 UniGene clusters. Many of these antisense transcripts were not observed previously and some were validated by orientation-specific RT-PCR.

**Conclusion:**

Our results suggest that with a modified protocol Affymetrix human, mouse and rat Exon arrays can be used as a routine method for genome-wide analysis of antisense transcription in these genomes.

## Background

Genome-wide analyses of mRNA and cDNA sequences have revealed large-scale antisense transcription in various animal and plant species. Based on analysis of mouse full length cDNA generated by FANTOM2 consortium, Okazaki *et al*. identified 2,481 pairs of overlapping sense/antisense transcripts [1]. An updated analysis using FANTOM3 cDNAs and mouse cDNA at GenBank showed that there are 4,520 transcription units forming sense and antisense pairs on exons [2]. For human, Yelin *et al*. [3] and Chen *et al*. [4] reported 2,667 and 2,940 pairs, respectively. The prevalence of natural antisense transcripts (NATs) is also supported by data derived by sequence tag-based technologies such as serial analysis of gene expression (SAGE) [5-7] and massively parallel signature sequencing (MPSS) [8].

Microarray-based studies also confirmed the prevalence of antisense transcripts. Using strand-specific oligonucleotide probes Yelin *et al*. studied the expression of both strands of 264 genomic loci in 19 human cell lines and detected antisense transcription in 112 (42.5%) of these loci [3]. Kiyosawa *et al*. studied the expression of 1,947 mouse NAT pairs in five types of cells and tissues using custom-made oligonucleotide arrays that distinguish the expression of sense versus antisense transcripts [9]. A research group at Affymetrix [10,11] used a novel direct RNA end-labeling method to detect the orientation of transcripts with a tiling array integrating chromosome 21 and 22 at a resolution of every probe per 35 base pair (bp). The analysis was later extended to 10 chromosomes at 5 bp resolution [12]. The data strongly support the observation that transcripts encoded on both DNA strands often results in complementary mRNAs. By focusing on 1% of human genome, the ENCODE project generated tiling array data suggesting that the majority of the bases of human genome is transcribed [13].

To investigate potential functions of the large number of NATs clearly requires cost-efficient technology for genome-wide expression analysis in a strand-specific manner. Because of the lack of technology for routine analysis, an interesting study took advantage of incorrectly orientated probes in commercial arrays [14]. About 25% of the probes on the first version of Affymetrix mouse U74A and U74B arrays were designed and manufactured in the wrong orientation [15]. Werner *et al*. used these faulty arrays to detect the expression of antisense transcripts in mouse brain and kidney [14]. Their result show that the antisense transcription is tissue specific and that the sensitivity of commercial arrays is sufficient to assess NATs in total RNAs.

In this proof-of-concept study, we demonstrate the applicability of Affymetrix Exon array to detect antisense transcripts at the whole-genome level. By modifying the standard cDNA synthesis and labeling process, we could labels single-strand cDNA in the reverse direction as compared to the standard protocol. Thus we can use Affymetrix Exon array to detect transcripts from antisense strands at over 1 million exonic loci across the human genome. Unlike previous expression arrays that target the 3' end of annotated genes, the Affymetrix Human Genome Exon array includes probes for all known and predicted exons. Most exons are represented by a probe-set consisting of 3 to 4 probes of 25 bp. For convenience, a 25 bp probe will be referred to as a "feature" and a probe-set will be called "probe" in the rest of the paper. We present a protocol that allows the independent labeling of sense and antisense strand of an RNA sample in combination with exon arrays will bring routine examinations of the antisense transcriptome within reach.

## Results

The Affymetrix Exon array is an inexpensive high-density oligonucleotide microarray that has two unique features: (1) it includes probes for all known and predicted exons, and (2) its signals are strand-specific because of the generation and labeling of single-stranded DNA targets. Exon arrays are currently available for human, mouse, and rat. In the standard protocol [16], an initial cycle of reverse transcription (RT) converts RNA into cDNA using random primers linked with the T7 promoter. This is followed by second-strand cDNA synthesis. The double-stranded cDNA is then used as a template for *in vitro *transcription (IVT) with T7 RNA Polymerase, which produces many copies of cRNAs that are reverse complementary to original RNA molecules. In the second cycle of cDNA synthesis, random primers are used to reverse transcribe the cRNA to obtain single-stranded DNA. The DNA is then fragmented and labeled in preparation for hybridization. After two RT cycles, the final single-strand DNA product is in the same orientation as original RNA. Based on annotation databases, the probes have been pre-manufactured to be reverse complementary to RNA sequences, so that the labeled product could hybridize with them.

We have tested a modified protocol called Antisense Transcriptome analysis using Exon array (ATE). Compared with the standard protocol outline above, ATE skips the first cycle cDNA synthesis and the IVT process. The ATE protocol starts directly from the second cycle cDNA synthesis in the standard protocol, where RNA sample is used as a template to synthesize single-strand cDNA through RT with random primers. The cDNA is then fragmented and labeled as recommended. Since only one cycle of RT is involved, the labeled cDNA fragments are reverse complementary to original RNA molecule, in contrary to the standard protocol. When hybridized to Exon arrays, cDNA derived from annotated genes can no longer hybridize to the probes since they are in the same orientation. Instead, if there is any transcript from the opposite DNA strand of the same genomic loci, they will give rise to cDNA sequences reverse complementary to the probes, thereby producing signals. Therefore, hybridization signals will represent antisense transcripts, instead of the intended sense transcript.

To test the ATE protocol, total RNA of Jurkat cells was used for both sense and antisense analysis. After removal of ribosomal RNA (rRNA) from 100 μg total RNA, we obtained 13.65 μg mRNA-enriched RNA. Sense strand expression profiling was performed according to standard protocol using 250 ng of this RNA. A larger amount (12 μg) was used to study the antisense gene expression according to our ATE protocol (see Methods for details). This will compensate for the skipped IVT amplification step and ensure sufficient yield of labeled targets. In the antisense array, we obtained 6.4 μg fragmented, labeled cDNA, of which 6 μg was used for hybridization.

The Affymetrix Expression Console software were used to normalize CEL files using the quantile normalization method and then summarize data at both the exon level and gene level by using the Robust Multichip Analysis (RMA) algorithm [17]. For comparison, CEL files of the 73 Exon array were downloaded from Affymetrix website, normalized and analyzed together. These 73 arrays represent 11 types of normal tissues and some colon tumor samples. Examination of quality control metrics showed that both sense and antisense array data were of high quality.

**Figure 1:**
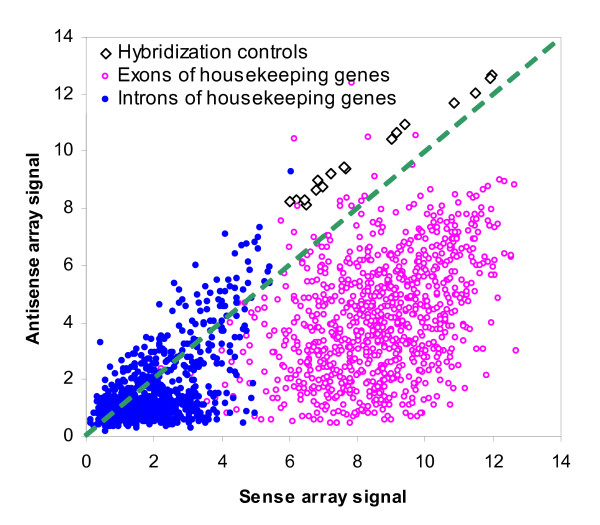
**Scatter plot of the signals of control probes in sense (x-axis) and antisense (y-axis) arrays**. The hybridization control probes (open squares) are highly expressed in both arrays. The signals of introns of housekeeping genes (blue dots) are low in both arrays at comparable levels. The signals of exons of housekeeping genes (open circles in red), however, are smaller by two orders of magnitude in the antisense array, indicating that antisense labeling does not mark sense transcripts. Signals are in log scale with base 2.

The hybridization-control probes produced signals at compatible levels in both arrays, although signals in antisense array were higher (Fig. 1). Independent of the expression signal, a detection P value was derived for each probe by using the DABG (Detected Above BackGround) algorithm to indicate whether the signal is significantly higher than 1000 negative control probes with similar GC content. In the sense array, 41.4% of 1.38 million probes are called "present" with a detection P value less than or equal to 0.01. The percentage of "present" probes in the antisense array is much smaller (13.4%). Unlike previous Affymetrix GeneChip arrays, the Exon array lacks mis-match probes to serve as probe-specific negative controls. This makes the "present" calls less reliable and thus we could not assume that 13.4% of exonic loci give rise to antisense transcripts. As discussed in the following sections, other measures should be used to complement detection calls to rule out non-specific signals.

Since fewer genes were expressed in the antisense array, quartile normalization led to larger signals. The hybridization control probes in the antisense array produced signals that were on average 1.58 times larger than these in the sense array. In order to make signals compatible, we linearly scaled down the antisense signal by this factor.

### Signals of control probes indicate that antisense array is strand-specific

The Affymetrix exon array includes a set of 1195 positive control probes representing exons of 100 housekeeping genes that are usually highly expressed in most tissues. The array also includes 2904 negative-control probes. They are selected from the intronic regions of these genes that are not supposed to be expressed. Based on the sample dataset provided by Affymetrix covering 11 types of normal tissues, we filtered out some positive controls that fail to produce comparably high expression scores across all samples and some negative controls produce high scores in some or all of the samples. The numbers of positive and negative control probes were reduced to 915 and 942, respectively.

Figure 1 shows the expression of these probes in both sense and antisense arrays. The signals from negative control probes in both arrays are at the same low levels. On the other hand, the 915 positive control probes gave significantly higher signals in the sense array than in the antisense array. Also, within the sense array, positive control probes produce signals that are about two orders of magnitude higher than negative control probes. In the antisense array this strong induction is diminished as both positive and negative controls produce very low signals. This is expected since the 915 positive control probes are designed to detect high signals only on the annotated strand. Antisense transcription is not likely to occur on these loci of housekeeping genes because of their high expression level and fundamental importance for basic cellular function. The low signals of positive control probes indicate that our ATE approach does not detect transcripts on the sense strand, including housekeeping genes expressed at high levels.

### Known antisense transcripts are detected with strong signals

**Figure 2:**
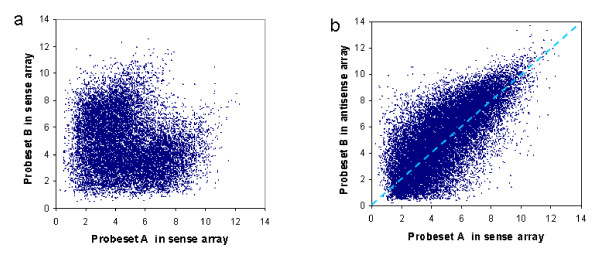
**Expression of sense-antisense probe pairs**. Each point represents a pair of probes, A and B, which target different strands of the same genomic loci. Shown in the left (**a**) are the expression levels of probe A and B in the same (sense) array. The right figure (**b**) gives the expression level of probe A in sense array (x-axis) versus that of probe B in the antisense array (y-axis). Signals are in log scale with a base of 2.

The Affymetrix Exon array includes pre-designed probes for some known antisense transcripts. We identified 24,750 pairs of probes that form sense-antisense pairs. We first searched for all pairs of probes whose targeted genomic region overlaps with each other, and then selected those pairs that are on opposite DNA strands. As shown in Fig. 2A, on the same array, these pairs have a tendency toward negative correlation in their expression (Pearson's correlation coefficient R = -0.18). The "L"-shaped scatter plot indicates co-expression of many of these pairs, but high expression (log2 signal > 6) of both strands is not observed. Between the sense and antisense arrays (Fig. 2B), these pairs showed a strong positive correlation (R = 0.72). This is expected since the same transcript is detected by a probe in the sense array and by an adjacent probe on the opposite strand in the antisense array. This strong positive correlation serves as a positive control for our approach.

**Figure 3:**
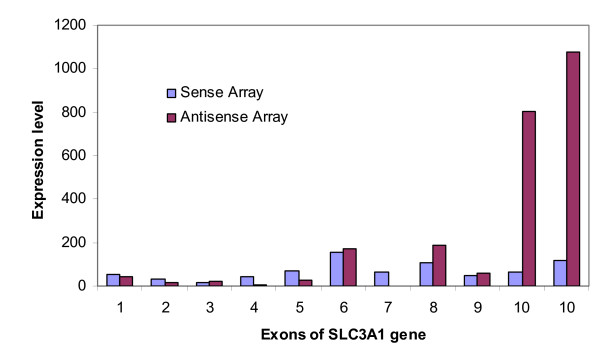
**The expression levels of the 10 exons of the SLC3A1 gene on Chr.2p21**. The expression levels of the exons are very low as measured by the sense array processed according to standard protocol. A strong signal is detected by the antisense array on the last exon, indicating transcription from this exon on the opposite strand.

On the level of individual genes, many known NATs were detected by our ATE method. For example, Fig. 3 gives the expression levels of all the exons of the solute carrier family 3, member 1 (SLC3A1) gene. The sense array suggests a very low level of expression across all exons. Examination of detection P values indicates that that expression of all these exons is too low to be detectable via microarray analysis. In other words, the signals are indistinguishable from the background defined by a set of negative control probes matched by GC content. The antisense array, however, detected strong signals in the two probes representing the last exon. This could suggest the expression of NATs on this exon.

Also notice that for exons No.1 to No. 9, the signals of sense and antisense arrays are approximately equal for the same probe, despite the fact that they are detecting transcripts on different strands. The correlated signals indicate probe-dependent non-specific signal. Compared with these nine probes, the 10-fold increase of exon 10 in the antisense strand is remarkable.

**Figure 4:**
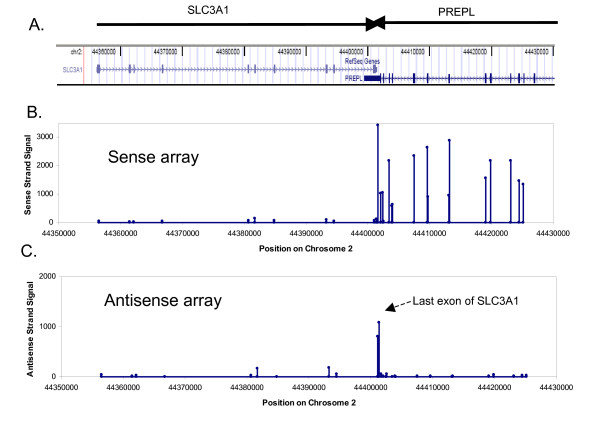
**Expression data for a gene with a known antisense transcript**. (**A**) The 3' ends of *SLC3A1 *and *PREPL *genes overlap on their last exons. (**B**) On the sense array, exons of *PREPL *gene are highly expressed while all exons of the *SLC3A1 *gene are extremely low. (**C**) Processed according to our ATE protocol, the antisense array only detects higher levels of expression of the last exon of the *SLC3A1 *gene.

The SLC3A1 gene is known to form sense-antisense pair with another well-annotated gene prolyl endopeptidase-like (*PREPL*) [3,4]. These two genes overlap on their last exons and are transcribed from different DNA strands (Fig. 4A). The *PREPL *gene is apparently highly expressed in Jurkat cell, because most exons of this gene are detected with high signals (Fig. 4B). The strong signal (Fig. 4C) picked up by the antisense array at the last exon of the *SLC3A1 *gene thus must come from its antisense genes, *PREPL*. Therefore, the antisense transcript of *SLC3A1 *is successfully detected. Also note that the exons of the *PREPL *gene were not detected by the antisense array. This reassured us again of the strand-specificity of our method.

Our ATE method also detected many other similar pairs such as {*NIT1*, *DEDD*} pair on Chr. 1q21, {*ADCY3*, *CENPO*} pair on Chr. 2p24, etc. Although we are more interested in cases in which the antisense transcript is not well annotated, detection of known antisense transcripts serve as a strong confirmation of our ATE protocol.

### Thousands of antisense transcripts are detected

To determine the number of antisense transcripts confidently detected in Jurkat cells by our ATE approach, we performed a series of filtering procedures. First of all, we focused on exons supported by full-length mRNAs like RefSeq. This reduced the number of probes from over 1 million to 289,961. Probes that are represented by less than 3 features or probes that could cross-hybridize to other transcripts were also eliminated. The remaining 184,163 high-quality probes will be analyzed in both sense and antisense strands.

To define genes confidently detected by antisense array, we first required that the signal to be higher than those of the 942 negative control probes on the same array. Background levels are indeed probe-specific, but most variations can be contained within a certain range and an absolute cutoff value is still useful. A threshold of log2-based score of 4.34 was chosen as only 29 (3.0%) of these probes are above this level. Also the detection P value should be smaller than 0.01. To further eliminate non-specific signals, the signal produced by a probe in the antisense array must be higher than the signals produced by the same probe in most of the 73 Exon arrays. Since Affymetrix exon arrays do not include mis-match probes that serve as probe-level controls, we found defining a probe-specific background signal by the distribution of signals in a large number of hybridizations very important. For each probe we calculated the mean and standard deviation of the signals observed in the 73 samples, which were used to compute a Z score to represent the observed signal in antisense or sense array. We used a conservative threshold of a Z score larger than 1, which is approximately equal to the requirement that the observed signal should be larger than 62 (86%) of 73 samples in the Affymetrix dataset.

Using these criteria, we determined the number of expressed probes in both arrays. As shown in Table 1, the number of probes detected by the antisense array (2,282) is much smaller than that detected by the sense array (39,373). The 2,282 probes detected by the antisense array represent 2,088 exons of 1,516 UniGene clusters. These probes and expression data are available in additional file 1. Hence, antisense transcripts targeting 1,516 well-annotated genes were detected by our method. A relatively large proportion (38.5%) of the exons targeted by antisense transcripts represents untranslated regions (UTRs). Among 1,516 genes, 490 (31.6%) have been reported by Chen *et al*. [4] or Yelin *et al*. [3] that were involved in antisense transcription. In other words, we identified 1,026 potentially novel antisense transcripts that were not detected by studies based on public databases of expressed sequences. If the 4,070 unique pairs reported by Chen *et al*. [4] or Yelin *et al*. [3] are considered the set of known antisense transcrits, we detected the expression 12% (490 out of 4,070) of them in Jurkat cells.

**Table 1:** Detected exons in Jurkat cells in sense and antisense arrays.

	Probes	Exons	Transcript Clusters	UniGene Clusters
Total on array	1,384,231	1,084,639		
Highly specific probes for known genes	184,163	143,243	17,605	16,332
Expressed in Jurkat cell (sense array)	39,373	33,639	6,469	6,205
Expressed in Jurkat cell (antisense array)	2,282	2,088	1,718	1,516
Expressed in both arrays (% of antisense)	392 (17%)	430 (21%)	652(38%)	585(39%)

**Figure 5:**
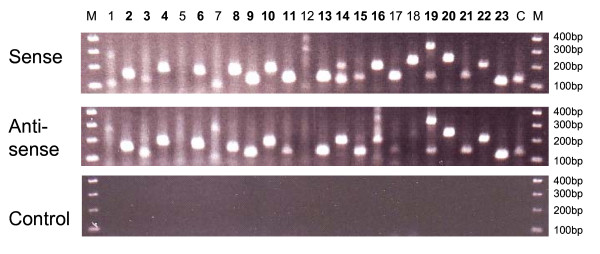
**Validation of antisense transcripts by orientation-specific reverse transcription (RT) PCR**. A total of 23 novel antisense transcripts detected by microarray were included. In addition, one known sense-antisense pair (*ADCY3 *and *CENPO*), which is marked as "C", was included as a positive control. For each genomic locus, three RT reactions were performed: (a) RT with reverse primer, targeting sense transcript (b) RT with forward primer, targeting antisense transcript, and (c) RT without primer, as negative control. Bolded primer IDs indicate bands that were considered significant in antisense transcription.

Using orientation-specific RT-PCR we performed validation of 24 of the identified NATs (see Fig. 5) according to the protocol of Chen *et al*. [4]. One known sense-antisense pair corresponding to two genes (ADCY3 and CENPO) with overlapping 3' UTR was included and was confidently detected. The negative control was performed by reverse transcription in absence of primers followed by PCR amplification. No signal was detected across all 24 cases, which excludes genomic DNA contamination and primer independent cDNA synthesis [18]. Strong bands suggest that 17 (74%) of 23 identified novel antisense transcripts, in agreement with our antisense microarray results.

Since Affymetrix Exon array includes probes interrogating both strands of some genomic loci, it is also possible to detect some antisense transcripts based on data from the sense array alone. Applying the above-mentioned criteria to pairs of overlapping probes on both strands, we detected antisense transcripts targeting 649 genes. Over half (52.5%) of these genes have been reported to form sense-antisense pairs in refs. [3] or [4]. Therefore, detection of antisense transcripts using these probes is limited by available annotation, whereas our new method can potentially detect antisense transcripts on all exons across the human genome.

## Discussion

Our method is clearly limited to NATs at known or predicted exonic loci. A more comprehensive survey would use genomic tiling arrays in a strand-specific fashion. Currently, the Affymetrix Human Tiling 2.0R array set uses seven chips to interrogate the non-repetitive regions of the whole genome at a 35 base pair resolution. Thus the cost of tiling array is much higher and use of tiling arrays for routine measurements of NAT expression in a large number of samples is difficult. Exon array provides a good balance between cost and coverage, as it targets most transcriptionally active loci at an affordable price.

The elimination of the RNA amplification process necessitates a large amount of total RNA. This is clearly a limitation of the ATE method. Our antisense array hybridization used about ~80 μg of total RNA. This does not translate into a huge amount of tissues. Even for the RNA-poor tissues like muscle and lung, this only requires no more than 80 mg of tissue. RNA yields for other common tissues are often 2 to 5 times higher [19]. Therefore, in addition to cell lines and normal tissues, ATE method should be applicable to many clinical studies for *simultaneous monitoring of transcriptions at both sense and antisense orientatio*n. In addition to human exon array, Affymetrix also provides similar Exon arrays for mouse and rat. In these model animals RNA is often available in large quantities, and the ATE method could be valuable for large-scale study of antisense expression.

## Conclusion

We demonstrated that high-throughput expression analysis of antisense transcripts could be achieved by using commercial DNA microarrays. By modifying the recommended cDNA synthesis protocol, it is possible to label targets in reverse orientation as what would be labeled according to the standard protocol. Our microarray data on human Jurkat cells showed that the modified protocol can successfully detect a large number of NATs transcribed from known exonic loci.

## Methods

### Materials

Total RNA (100 μg, Cat.#7858) of human Jurkat cells (derived from T-cell leukemia) was purchased from Ambion (Austin, TX). Ribosomal RNA (rRNA) reduction was performed using the RiboMinus Kit from Invitrogen (Carlsbad, CA). This sample was used for both sense and antisense analyses.

### Sense strand expression analysis using standard protocol

Sense strand expression profiling was performed according to recommended protocol [16]. Briefly, double-stranded cDNA is synthesized with random hexamers coupled with a T7 promoter sequence. The cDNA is then used as a template for *in vitro *transcription (IVT) amplification with T7 RNA Polymerase, producing multiple copies of cRNAs that are reverse complementary to original mRNA. In the second cycle cDNA synthesis, random primers are used in reverse transcription to convert the cRNA into single-stranded DNA in the same orientation as original mRNAs. The single-stranded cDNA are then fragmented, labeled, and hybridized to the array. To ensure hybridization, the probe sequences on the array are pre-manufactured in the opposite orientation of mRNAs registered in annotation databases.

### Antisense Transcriptome analysis using Exon (ATE) array

Compared with standard protocol, the ATE protocol skips the first cycle cDNA synthesis and the IVT amplification process, and starts directly from the second cycle cDNA synthesis. The labeled target DNA fragments are in reverse orientation of original mRNAs. Thus hybridization signals will represent transcripts from the same exonic regions but from the opposite DNA strand.

Briefly, the ATE protocol involves the following steps:

1. Removal of ribosomal RNA from total RNA using the RiboMinus Kit.

2. 1^st ^strand cDNA synthesis using random primers. dUTP is incorporated in the DNA during reverse transcription.

3. Cleanup of RNA using RNase H.

4. The single-stranded DNA sample is treated by a combination of uracil DNA glycosylase (UDG) and apurinic/apyrimidinic endonuclease 1 (APE 1) that specifically recognizes the dUTP residues and breaks the DNA into fragments.

5. Fragmented single-stranded DNA is labeled with recombinant terminal deoxynucleotidyl transferase (TdT) and the Affymetrix DNA Labeling Reagent. The reagent is covalently linked to biotin.

6. Hybridization and scanning.

The ATE protocol is a straightforward method for measuring antisense transcription without involving double-strand DNA. For details on each of the above steps, see [16].

### Validation of antisense transcripts using strand-specific RT-PCR

Total RNA from Jurkat cell (Ambion, TX) was used as template, which was pre-treated with DNase I to remove potential genomic DNA contamination. Twenty four Pairs of PCR primers were designed using Primer3 software [20] to target 100–200 bp regions at 24 exonic loci that were found to have antisense transcription by our antisense array (see additional file 2 for the primers). We used a Qiagen One Step RT-PCR kit following a procedure similar to the one used by Chen *et al*. [4]. The strand-specificity of the Qiagen kit for studying antisense transcription is also confirmed by Haddad *et al *[18].

The experimental confirmation for each of the 24 NATs involves a reverse transcription (RT) step followed by a PCR reaction. Orientation of transcript was assessed by selective use of primers during single-strand cDNA synthesis in the RT step. To detect the sense transcripts, the reverse primer was used for RT; to detect the antisense transcript, the forward primer was used for RT. In addition, a negative control against genomic contamination was carried out by performing RT without any primer in RT. Therefore, three separate reactions were performed for each targeted NAT. All of these RT processes were followed by PCR reactions under identical conditions with the presence of both forward and reverse primers. PCR products were visualized on 2% agarose gels. The cycling parameters were: (1) 50°C × 30 m, reverse transcription for single-strand cDNA synthesis; (2) 95°C × 15 m, activate AmpliTaq polymerase, inactivate RT enzymes; (3) 4°C, add missing primers for PCR; (4) 94°C × 30s, commence PCR cycling; (5) 60°C × 30s; (6) 72°C × 60s; (7) go to step (4) and repeat 35 cycles in total; and (9) 72°C × 10 m. These cycling parameters are the same as those used by Chen *et al*. [4], except step (6) which was changed to 72°C × 60 s instead of 72°C × 35s.

## Authors' contributions

XG, WSR and QW conceived the study and designed the experiments. YJ performed experimental confirmation. XG did data analysis and wrote the manuscript. All authors have read and approved the final version of the manuscript.

## Supplementary Material

Additional file 1 List of detected antisense transcripts in Jurkat cells. This file lists the probes that were found to be confidently expressed in Jurkat cells at the antisense orientation. Both annotation and expression data are given. Click here for file

Additional file 2 List of PCR primers used for experimental validation. This file contains the forward and reverse primers for 24 PCR reactions. Click here for file
